# Mass balance and pharmacokinetics of an oral dose of ^14^C‐napabucasin in healthy adult male subjects

**DOI:** 10.1002/prp2.722

**Published:** 2021-02-11

**Authors:** Xiaoshu Dai, Michael D. Karol, Matthew Hitron, Marjie L. Hard, John Evan Blanchard, Nicola C. J. E. Eraut, Natalie Rich, Brandon T. Gufford

**Affiliations:** ^1^ Sumitomo Dainippon Pharma Oncology, Inc. Cambridge MA USA; ^2^ Nuventra Inc. Durham NC USA; ^3^ Covance, Inc. Madison WI USA; ^4^ Covance Clinical Research Unit Ltd. Leeds UK; ^5^ Present address: Former employee of Nuventra Inc. Durham NC USA

**Keywords:** administration, oral, clinical trial, phase I, drugs, investigational, healthy volunteers, metabolism, pharmacokinetics

## Abstract

This phase 1, open‐label study assessed^14^C‐napabucasin absorption, metabolism, and excretion, napabucasin pharmacokinetics, and napabucasin metabolites (primary objectives); safety/tolerability were also evaluated. Eight healthy males (18–45 years) received a single oral 240‐mg napabucasin dose containing ~100 μCi^14^C‐napabucasin. Napabucasin was absorbed and metabolized to dihydro‐napabucasin (M1; an active metabolite [12.57‐fold less activity than napabucasin]), the sole major circulating metabolite (median time to peak concentration: 2.75 and 2.25 h, respectively). M1 plasma concentration versus time profiles generally mirrored napabucasin; similar arithmetic mean half‐lives (7.14 and 7.92 h, respectively) suggest M1 formation was rate limiting. Napabucasin systemic exposure (per C_max_ and AUC) was higher than M1. The total radioactivity (TRA) whole blood:plasma ratio (AUC_last_: 0.376; C_max_: 0.525) indicated circulating drug‐related compounds were essentially confined to plasma. Mean TRA recovery was 81.1% (feces, 57.2%; urine, 23.8%; expired air, negligible). Unlabeled napabucasin and M1 recovered in urine accounted for 13.9% and 11.0% of the dose (sum similar to urine TRA recovered); apparent renal clearance was 8.24 and 7.98 L/h. No uniquely human or disproportionate metabolite was quantified. Secondary glucuronide and sulfate conjugates were common urinary metabolites, suggesting napabucasin was mainly cleared by reductive metabolism. All subjects experienced mild treatment‐emergent adverse events (TEAEs), the majority related to napabucasin. The most commonly reported TEAEs were gastrointestinal disorders. There were no clinically significant laboratory, vital sign, electrocardiogram, or physical examination changes. Napabucasin was absorbed, metabolized to M1 as the sole major circulating metabolite, and primarily excreted via feces. A single oral 240‐mg dose was generally well tolerated.

## Graphical abstract

Proposed biotransformation pathways of napabucasin in humans. A single 240‐mg oral dose of napabucasin was extensively metabolized to produce 30 metabolites, all of which were found in urine, five in plasma, and one in feces, in healthy male subjects. Based on these results, we propose a biotransformation pathway of napabucasin in humans: reduction of the acetyl side‐chain and/or the naphthalene dione moiety is the exclusive primary biotransformation pathway, with glucuronidation, sulfonation, and, to a lesser extent, transamination as abundant secondary routes of metabolism.

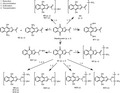

**Abbreviations**
AE: adverse event
AUC: area under the curve
ECG: electrocardiogram
MS: mass spectrometry
PK: pharmacokinetics
TEAE: treatment‐emergent adverse event
TRA: total radioactivity

## 1 INTRODUCTION

Napabucasin is an orally administered reactive oxygen species (ROS) generator bioactivated by the intracellular antioxidant NAD(P)H:quinone oxidoreductase 1 (NQO1).^1^, ^2^ Napabucasin exerts its antitumor activity by increasing levels of ROS beyond a cytotoxic threshold, causing cancer cell death.^1^, ^2^, ^3^ Treatment of several cancers with napabucasin alone or in combination with other cancer therapies has been assessed in a number of ongoing clinical trials (congress abstracts,^4^, ^5^, ^6^, ^7^, ^8^, ^9^, ^10^, ^11^, ^12^, ^13^, ^14^, ^15^, ^16^ and completed clinical trials^17^, ^18^). An ongoing phase 3 study is assessing napabucasin in combination with 5‐fluorouracil, leucovorin, and irinotecan (i.e., FOLFIRI) in patients with previously treated metastatic colorectal cancer (Clinicaltrials.gov identifier: NCT02753127).^19^

Preclinical studies of napabucasin suggest it is excreted via the renal as well as biliary/fecal routes in rats and mainly via the biliary/fecal route in dogs [Data on file, Sumitomo Dainippon Pharma Oncology, Inc.]. In vitro metabolic studies in cryopreserved hepatocytes from rats, dogs, rabbits, and humans showed that napabucasin metabolites were qualitatively similar among species [Data on file, Sumitomo Dainippon Pharma Oncology, Inc.].

The primary objectives of the current study were to characterize the absorption, metabolism, and excretion of ^14^C‐napabucasin, and to determine the pharmacokinetics (PK) of napabucasin and relevant metabolites in plasma, urine, and feces. Secondary objectives were to assess the safety and tolerability of napabucasin in healthy male subjects following a single oral dose of 240‐mg napabucasin.

## 2 MATERIALS AND METHODS

### 2.1 Study design

**FIGURE 1:**
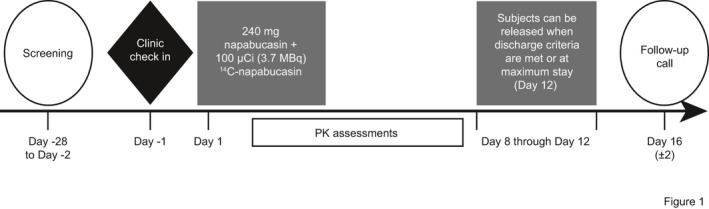
Study Design. Healthy male subjects received a single oral dose of 240‐mg napabucasin containing approximately 100 μCi of ^14^C‐napabucasin in a phase 1, open‐label study (NCT03525405). PK, pharmacokinetic

This was a phase 1, open‐label, single‐dose study (NCT03525405) conducted in healthy male subjects who received a single oral dose of 240‐mg napabucasin containing approximately 100 μCi of ^14^C‐napabucasin (Figure 1). This study was conducted in compliance with the ethical principles of the International Conference on Harmonization, Good Clinical Practice guidelines, all federal, state, and local laws, and in accordance with the Declaration of Helsinki. The protocol was approved by the Institutional Review Board at the study site, and all subjects provided written informed consent prior to participation in this study.

### 2.2 Subjects

Healthy male subjects between the ages of 18 and 45 years and with a body mass index between 18 and 34 kg/m^2^ were eligible for inclusion. Good health was defined as no clinically significant findings from medical history, physical examination, 12‐lead electrocardiogram (ECG), vital signs measurements, or clinical laboratory evaluations. Key exclusion criteria included a history of illicit drug abuse within the past year; positive finding on a urine drug screen; positive serostatus for human immunodeficiency virus, hepatitis B virus, or hepatitis C virus; alcohol consumption for 3 days prior to clinical research unit (CRU) admission; use of tobacco‐ and nicotine‐containing products for 2 months prior to CRU admission; and consumption of dietary supplements, herbal products, nonprescription drugs, and prescription drugs (except as authorized by the Investigator and Medical Monitor) for 14 days prior to CRU admission. Subjects were required to abstain from consumption of alcohol, tobacco/nicotine, and all medications or supplements during the study and through the follow‐up call. Enrollment of eight healthy male subjects was planned to ensure at least six subjects completed the study.

### 2.3 Study procedure

Figure 1 shows a schematic of the study procedure. Subjects were confined at the CRU from the time of check‐in (Day −1) until discharge (Day 8–12). On Day 1 of the Treatment Phase, following an overnight fast, a single 240‐mg dose containing approximately 100 µCi of ^14^C‐napabucasin was administered with 240 ml of water. Subjects remained fasted for 4 h after dosing. The study drug was delivered via three hard gelatin capsules. One of the capsules contained approximately 100 μCi (3.7 MBq) of ^14^C‐napabucasin (specific activity = 39.2 μCi/mg, chemical and radiochemical purity = 100%; Moravek Biochemicals, Inc.) blended with 80 mg of napabucasin; the remaining two capsules contained napabucasin only (for a total 240‐mg dose of napabucasin). For the radiolabeled capsule, radiolabeled napabucasin had to be mixed with the nonlabeled napabucasin, which is formulated as a semisolid suspension. Due to limited solubility, the radiolabeled napabucasin could not be homogeneously mixed with the semisolid suspension. This resulted in capsules with the same amount of radiolabeled material in each; however, consistent distribution of radiolabeled and nonlabeled napabucasin within the capsule could not be assured. Based on dosimetry projections, the administration of a single oral dose containing approximately 100 μCi (3.7 MBq) of ^14^C‐napabucasin was not expected to represent a significant radiation exposure risk in human male subjects. The FDA‐established radiation exposure limit is 3000 mrem (30 mSv).^20^ The estimated radiation absorbed for critical tissues (i.e., bone marrow, breast, lung, thyroid) ranges from 0.0000163 to 0.0557 mrem (0.000000163–0.000557 mSV; excluding bone and testes) based on the International Commission on Radiological Protection Publication 30. In addition, relatively low quantifiable radioactivity was detected in rat studies in bone, testes, and eye lens after a single ~100 μCi/kg ^14^C‐napabucasin dose, suggesting that radiation exposure in these tissues in humans would be well below 3000 mrem (30 mSv) (Data on file, Sumitomo Dainippon Pharma Oncology, Inc.). The highest absorbed radiation doses in humans were projected for the renal cortex, kidneys, renal medulla, large intestine, and epididymis (0.416–1.95 mrem; 0.00416–0.0195 mSv) (Data on file, Sumitomo Dainippon Pharma Oncology, Inc.). The overall estimated whole‐body effective dose was 0.293 mrem (0.00293 mSv) (Data on file, Sumitomo Dainippon Pharma Oncology, Inc.) . All projections of radiation absorbed by a single 100 μCi ^14^C‐napabucasin dose were substantially below the exposure limit established by the FDA.^20^

Subjects were to be discharged when >90% of radioactivity had been recovered and two consecutive 24‐h urine and fecal samples contained <1% of the radioactivity administered as indicated in a protocol amendment, or if the protocol‐specified maximum stay of Day 12 occurred. Subjects completed a follow‐up phone call on Day 16 (±2 days).

### 2.4 Sample collection

Whole blood, urine, and fecal samples were collected pre‐dose, at predefined points throughout the study until 168 h post‐dose, followed by every 24 h until discharge. Specifically, whole blood was collected via peripheral venipuncture—within 30 min prior to dose and at 0.5, 1, 1.5, 2, 2.5, 3, 4, 6, 8, 10, 12, 18, 24, 36, 48, 60, 72, 96, 120, 144, and 168 h post‐dose, followed by every 24 h until discharge—into lavender top collection Vacutainer^®^ tubes containing K_2_EDTA (Becton, Dickinson and Company). Whole blood was stored at −70°C for measurement of total radioactivity (TRA). For plasma preparation, blood samples were centrifuged at 1700*g* at 4°C for 10 min within 60 min of collection. Plasma was aliquoted and stored at −70°C. Urine was collected at pre‐dose (approximately −12 to 0 h), and 0–4, 4–8, 8–12, 12–18, 18–24, 24–36, 36–48, 48–72, 72–96, 96–120, 120–144, and 144–168 h post‐dose, followed by every 24 h until discharge. Urine samples were pooled for each collection period and weights were documented before storing at 4°C. Fecal samples were collected pre‐dose (−24 to 0 h), 0–24, 24–48, 48–72, 72–96, 96–120, 120–144, and 144–168 h post‐dose, followed by every 24 h until discharge, and stored at −70°C within 60 min. Fecal samples were combined for each subject at 24‐h intervals, weights were documented, and samples were homogenized with a weighed amount of water using a probe‐type homogenizer. Aliquots were stored at −70°C. Expired air was collected into clear 20‐ml scintillation vials at 5, 12, 24, and 48 h post‐dose, followed by subsequent 24‐h time points, as needed; samples were stored at −20°C.

### 2.5 Analytical methods

For TRA measurement, whole blood and fecal samples were combusted and analyzed by liquid scintillation counting. All other samples were analyzed directly by liquid scintillation counting. All urine and fecal samples were processed for determination of ^14^C‐radioactivity on a “real‐time” basis. Further details of the TRA measurements are provided in the supplemental data.

Concentrations of napabucasin and its currently known primary metabolite dihydro‐napabucasin (M1) were determined in plasma and urine using liquid chromatography‐tandem mass spectrometry (LC, Shimadzu SCL‐10A controller with LC‐10/20AD pump)–tandem mass spectrometry (MS/MS, Sciex API 6500 platform). Samples were separated on a 2.1 × 100 mm Luna C18(2) 100A, 5‐µm column (Phenomenex) with mobile phase A of 0.1% formic acid in water, mobile phase B of 0.1% formic acid in 75/25 MeOH/MeCN, mobile phase C of 0.1% formic acid in water, a flow rate of 0.6 ml/min, and gradient event sequence of 40% B at 0.01 min, divert to MS at 2.80 min, 55% B at 2.75 min, 95% B at 3.75 min, divert to waste at 4.10 min, 95% B at 4.30 min, 40% B at 4.5 min, and stop at 5.0 min. The calibration range was 5–5000 ng/ml in plasma and 100–15 000 ng/ml in urine for napabucasin and M1. Accuracy and precision values were within the acceptance criteria (Table S1).

The metabolic profile of ^14^C‐napabucasin was determined in plasma, urine, and feces by means of accelerator MS with liquid chromatography‐MS. Plasma samples were pooled across time points (individual subject area under the curve [AUC]_0.5–120 h_ pools) and across subjects (across‐subject AUC_0.5–120 h_ pool). Urine and fecal homogenate samples were pooled by subject. The across‐subject plasma AUC pool was profiled by accelerator MS by the Netherlands Organisation for applied scientific research as previously described.^21^ Individual AUC pools were analyzed by LC (Shimadzu Prominence/Nexera)‐MS (Thermo Fisher Scientific Q Exactive). The limit of quantitation for radioactivity was set at 1% of each chromatographic analysis (run) and 10 cpm peak height. The cut‐off for identification of metabolites was 1% of the radioactive dose for urine and feces. If collective excretion of the metabolites was greater than 10% of the TRA, then structural elucidation of metabolites was performed by comparison with known standards and LC‐MS. Detailed methods for measurement of metabolites are described in the supplemental data.

### 2.6 PK parameters

The PK parameters for ^14^C‐napabucasin and napabucasin were analyzed with Phoenix WinNonlin version 6.4 and version 8.1 (Certara USA, Inc.), respectively. Actual post‐dose times were recorded in the raw data and used in the estimation of PK parameters. PK parameters determined included AUC from time 0 to infinity (AUC_inf_), AUC from time 0 to time of last measurable concentration (AUC_last_), renal clearance (CL_R_), apparent systemic clearance (CL/F), peak concentration observed (C_max_), metabolite to parent AUC ratio (M/P‐AUC); metabolite to parent C_max_ ratio (M/P‐C_max_), ratio of analyte to TRA based on AUC (R‐AUC), ratio of analyte to TRA based on C_max_ (R‐C_max_), apparent terminal elimination half‐life (t_1/2_), lag time (t_lag_), time of last quantifiable concentration (T_last_), time to peak concentration (T_max_), and apparent volume of distribution (V_z_/F).

### 2.7 Safety assessments

Adverse events (AEs) were monitored and recorded throughout the study (treatment phase through follow‐up call). The nature, time of onset, duration, severity, and relationship to study drug were documented. Clinical laboratory evaluations, vital signs, 12‐lead ECGs, and physical examinations were assessed. Clinical laboratory evaluations (hematology, coagulation, clinical chemistry, and urinalysis) were assessed at screening, Day −1, and at clinical discharge. Hematology, coagulation, and clinical chemistry were also assessed on Day 2 of the treatment phase. Vital signs were assessed daily throughout the study from screening through clinical discharge. Twelve‐lead ECG was assessed at screening and at clinical discharge. Physical examinations were performed at Day −1 and at clinical discharge.

### 2.8 Statistical analyses

Data analysis was performed using SAS^®^ Version 9.4. For PK analysis, TRA in whole blood, plasma, urine, feces, and expired air, and napabucasin and M1 concentrations in plasma and urine were listed and summarized using descriptive statistics. Individual and mean plasma and whole blood concentration versus time profiles were presented graphically on both linear and semi‐logarithmic scales. Cumulative radioactivity excreted in urine and feces and total recovery versus collection interval were summarized and presented graphically utilizing the endpoint of the collection interval. Due to a lack of detectable radioactive material, expired air results were not summarized. For safety analysis, AEs were summarized using descriptive statistics. No inferential statistical analyses were performed.

## 3 RESULTS

### 3.1 Demographics and baseline characteristics

Overall, 10 subjects were screened and eight were enrolled and dosed (Table 1). Subjects were a mean age of 29 years (range: 23–39), black/African American (62.5%) or white (37.5%), with mean body mass index (BMI) of 26.3 kg/m^2^ (standard deviation [SD]: 4.0); all eight were analyzed for PK and safety. Two of the 10 subjects were screened as backups and were therefore not dosed.

**TABLE 1:** Baseline demographics

	All enrolled subjects (*N* = 8)
Age (years), mean (SD)	29 (5.1)
Male sex, *n* (%)	8 (100)
Race, *n* (%)	
Black or African American	5 (62.5)
White	3 (37.5)
Not Hispanic or Latino, *n* (%)	8 (100)
Weight (kg), mean (SD)	84.1 (13.3)
Height (cm), mean (SD)	178.8 (3.6)
BMI (kg/m^2^), mean (SD)	26.3 (4.0)

Abbreviations: BMI, body mass index; *N*, number of subjects; SD, standard deviation.

### 3.2 PK results

#### 3.2.1 Excretion

**FIGURE 2:**
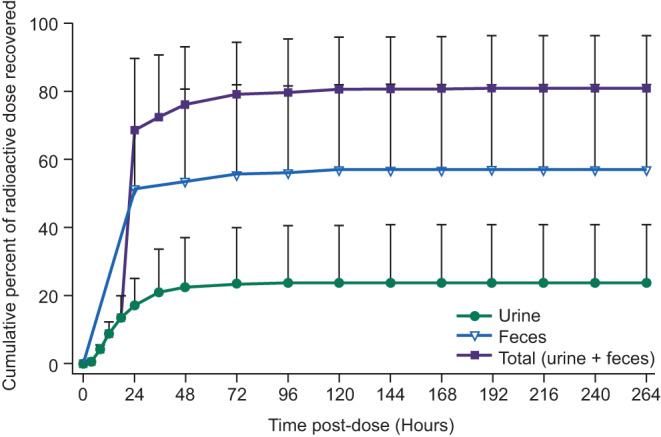
Cumulative excretion of radioactivity in urine and feces. Healthy male subjects received a single oral dose of 240‐mg napabucasin containing approximately 100 μCi of ^14^C‐napabucasin. TRA was measured in urine and feces collected at predefined points throughout the study until 168 h post‐dose. Data are reported for *N* = 8 and presented as mean ± standard deviation. TRA, total radioactivity

The mean TRA recovered in urine and feces was 81.1%. Most recovery (76.0%) was within 48 h post‐dose (Figure 2). TRA in urine was below the quantifiable limit at time points ranging from 48 to 192 h post‐dose. TRA in feces was below the quantifiable limit in five subjects at time points ranging from 72 to 216 h post‐dose. One subject had detectable fecal radioactivity at the last sampling interval (240–264 h post‐dose). The other two subjects were discharged from the study prior to 9 days (216 h) post‐dose; one had TRA below the limit of quantitation at 120–144 h post‐dose and the other had detectable fecal radioactivity at the last sampling interval (144–168 h post‐dose).

Excreted ^14^C‐napabucasin was predominant in feces (57.2%), followed by urine (23.8%), and was negligible in expired air (Table 2). In contrast with the arithmetic mean data, one subject excreted 62.8% and 27.7% of the dose in urine and feces, respectively. This subject had the highest plasma AUC_last_ and AUC_inf_ for napabucasin and M1 (~two‐fold greater than the median values), and higher AUC_last_ and AUC_inf_ for TRA in plasma and whole blood (where calculated), suggesting a greater dose absorption than in other subjects. Only two subjects had quantifiable TRA in expired air (0.000010% and 0.000017% recovery of administered dose).

**TABLE 2:** Urinary excretion of napabucasin and TRA recovered in urine and feces

Parameter	Napabucasin in urine	M1 in urine	TRA		
			Urine	Feces	Total
Total A_eu_ or A_ef_ (mg)	33.7 (12.6)	26.9 (17.3)	57.8^a^ (41.8)	139 (59.8)^a^	197 (37.6)^a^
Total F_eu_ or F_ef_ (%)	13.9 (5.18)	11.0 (7.06)	23.8 (17.2)	57.2 (24.8)	81.1 (15.6)
CL_R_ (L/h)	8.24 (1.70)	7.98 (1.53)	NA	NA	NA

Data are reported as arithmetic mean (SD) for *N* = 8.

TRA could represent labeled napabucasin, labeled M1, or other labeled napabucasin metabolites, which could vary over time, especially at late time points.

Abbreviations: A_ef_, amount excreted in feces from time 0 to last measurable concentration; A_eu_, amount excreted in urine from time 0 to last measurable concentration; CL_R_, renal clearance; CV, coefficient of variation; F_ef_, percent of dose excreted in feces from time 0 to last measurable concentration; F_eu_, percent of dose excreted in urine from time 0 to last measurable concentration; mg eq, milligram equivalents; NA, not applicable; SD, standard deviation; TRA, total radioactivity.

^a^ Presented in mg eq.

Napabucasin and M1 in urine accounted for 13.9% and 11.0% of the recovery of the administered dose, respectively (arithmetic means). The sum of the unlabeled napabucasin and M1 recovered in urine (24.9%) was similar to the percentage of radioactivity recovered in urine (23.8%), suggesting that napabucasin and M1 likely accounted for the majority of the material recovered via this route. This was also generally true for individual data. Both compounds were quantifiable in urine up to 24 h post‐dose, with the majority excreted in the first 8 h. Apparent renal clearance of napabucasin and M1 was similar, with arithmetic mean CL_R_ values of 8.24 L/h (range: 5.90–11.2) and 7.98 L/h (range: 5.74–10.4), respectively. This is consistent with the formation of M1 being rate limiting.

#### 3.2.2 Concentration‐time profiles

**FIGURE 3:**
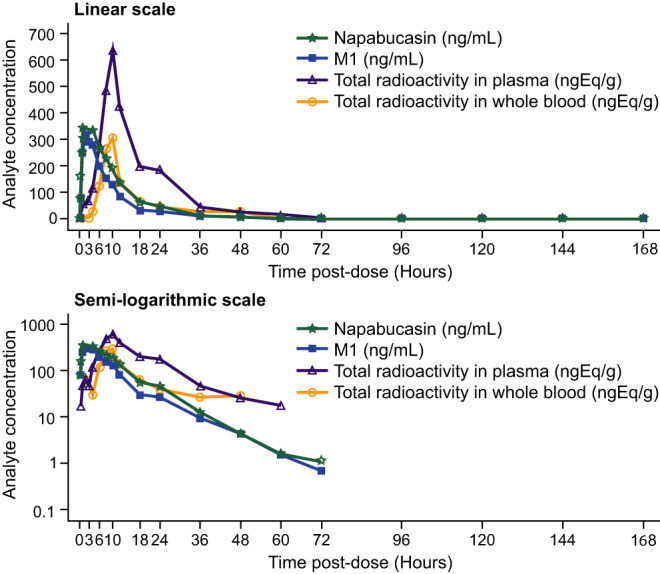
Mean concentration‐time profiles of napabucasin, M1, and TRA in plasma and whole blood. Healthy male subjects received a single oral dose of 240‐mg napabucasin containing approximately 100 μCi of ^14^C‐napabucasin. Blood samples were collected at predefined points throughout the study until 168 h post‐dose, followed by every 24 h until discharge. Napabucasin, M1, and TRA concentrations were measured. Data are reported for *N* = 8 and presented as arithmetic means. M1, dihydro‐napabucasin; TRA, total radioactivity

Napabucasin was absorbed with a median T_max_ of 2.75 h (range: 1–8). M1 was quantifiable in the first post‐dose time point for all subjects and attained median T_max_ at 2.25 h (range: 1.5–10). After reaching C_max_, individual plasma concentrations of napabucasin and M1 declined in a monophasic or biphasic manner with a similar arithmetic mean t_1/2_, 7.92 and 7.14 h, respectively. The similar half‐lives suggest that the formation of M1 was rate limiting. Napabucasin and M1 remained quantifiable until 24–72 h and 18–72 h post‐dose, respectively (median T_last_: 30 and 24 h, respectively). Generally, the plasma concentration‐versus‐time profile of M1 mirrored that of parent napabucasin. Exposure of M1 was generally lower than that of napabucasin, with arithmetic mean metabolite to parent C_max_, AUC_last_, and AUC_inf_ ratios of 0.844, 0.753, and 0.744, respectively (Table 3, Figure 3).

**TABLE 3:** Summary of plasma pharmacokinetics parameters

Parameter	^14^C‐napabucasin 240 mg			
	Napabucasin in plasma	M1 in plasma	Plasma TRA	Whole blood TRA
C_max_ (ng/ml or ng eq/g)	444 (127)	378 (147)	664 (398)	344 (196)
T_max_ (h)^a^	2.75 (1.00–8.00)	2.25 (1.50–10.00)	10.00 (4.00–12.00)	9.00 (6.00–10.00)
t_lag_ (h)^a^	0.00	0.00	1.25 (0–4.00)	4.00 (3.00–6.00)
AUC_last_ (h*ng/ml or h*ng eq/g)	4020 (1190)	3050 (1300)	7770 (6610)	3760 (2780)
AUC_inf_ (h*ng/ml or h*ng eq/g)	4200 (1180)	3160 (1300)	NC^b^	NC^d^
t_1/2_ (h)	7.92 (3.07)	7.14 (3.18)	11.8 (3.46)^c^	NC^d^
V_z_/F (L)	685 (282)	—	NC^b^	NC^d^
CL/F (L/h)	61.3 (15.1)	—	NC^b^	NC^d^
M/P‐AUC_last_	NA	0.753 (0.184)	—	—
M/P‐AUC_inf_	NA	0.744 (0.170)	—	—
M/P‐C_max_	NA	0.844 (0.244)	—	—
R‐AUC_last_	0.778 (0.529)	0.594 (0.461)	—	—
R‐AUC_inf_	0.464 (0.227)	0.382 (0.153)	—	—
R‐C_max_	0.972 (0.744)	0.844 (0.662)	—	—

Data are reported for *N* = 8. Arithmetic mean (SD) data are presented, except for T_max_ and t_lag_.

TRA could represent labeled napabucasin, labeled M1, or other labeled napabucasin metabolites, which could vary over time, especially at late time points.

Abbreviations: AUC, area under the curve; AUC_inf_, AUC from time 0 to infinity; AUC_last_, AUC from time 0 to time of last measurable concentration; CL/F, apparent systemic clearance; C_max_, peak concentration observed; CV, coefficient of variation; eq, equivalent; M/P‐AUC, metabolite to parent AUC ratio; M/P‐C_max_, metabolite to parent C_max_ ratio; NA, not applicable; NC, not calculated; R‐AUC, ratio of analyte to TRA based on AUC; R‐C_max_, ratio of analyte to TRA based on C_max_; SD, standard deviation; t_1/2_, apparent terminal elimination half‐life; t_lag_, time prior to first quantifiable concentration; T_max_, time to peak concentration; TRA, total radioactivity; V_z_/F, apparent volume of distribution.

^a^ Median (range) data are presented.

^b^ It was not possible to calculate the full summary statistics because a t_1/2_ estimate was not always possible (see footnote c) and was more than 30% extrapolated for 2 of 4 subjects.

^c^ Half‐life estimates were possible for 4 subjects, but for 3 subjects the estimates were determined over a period <2 half‐lives.

^d^ Terminal phase could not be defined for any profile in whole blood, so only observed parameters were calculable.

**FIGURE 4:**
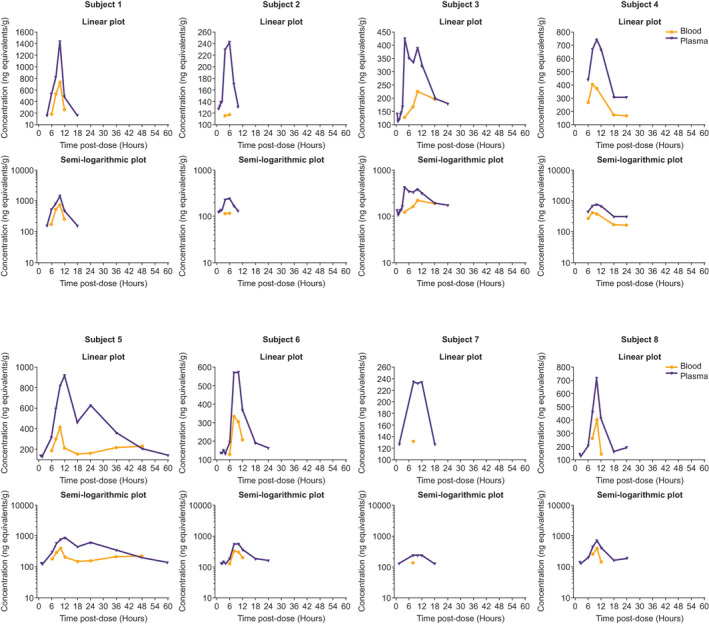
Concentration‐time profiles of TRA in plasma and whole blood of individual subjects. Healthy male subjects received a single oral dose of 240‐mg napabucasin containing approximately 100 μCi of ^14^C‐napabucasin. Blood samples were collected at predefined points throughout the study until 168 h post‐dose, followed by every 24 h until discharge. Napabucasin, M1, and TRA concentrations were measured. M1, dihydro‐napabucasin; TRA, total radioactivity

In contrast to cold napabucasin and M1, there was a delay in the absorption of TRA (median t_lag_ of 1.25 h [range: 0–4] in plasma and 4 h [range: 3–6] in whole blood). The median T_max_ for TRA in plasma was 10 h (range: 4–12) and for whole blood was 9 h (range: 6–10). TRA remained quantifiable in plasma and whole blood until 10–60 h (median T_last_ of 24 h) and 6–48 h (median T_last_ of 12.1 h), respectively (Table 3, Figure 3). Individual TRA profiles in plasma and whole blood were characterized by multiple peaks, which confounded the definition of a terminal phase (Figure 4). Of note, TRA could represent labeled napabucasin, labeled M1, or other labeled napabucasin metabolites, which could vary over time, especially at late time points. The difference in the concentration‐time profiles of TRA compared with unlabeled napabucasin and M1 was attributed to the heterogeneity of the dose, resulting in a differential rate and extent of absorption.

There was moderate to high intersubject variability for napabucasin and M1, and high intersubject variability for TRA, reflected by the moderate to high SDs for C_max_ and AUC_inf_ (Table 3).

Drug‐related material was essentially confined to the plasma compartment with arithmetic mean TRA whole blood:plasma ratio of 0.376 (AUC_last_) and 0.525 (C_max_). The arithmetic mean ratios of plasma napabucasin to TRA were 0.972, 0.778, and 0.464; and of plasma M1 to TRA were 0.844, 0.594, and 0.382, for C_max_, AUC_last_, and AUC_inf_, respectively. For plasma napabucasin and M1, the ratio of parent or M1 concentration to TRA based on C_max_ and AUC_last_ was >1 for some individuals, which is also attributed to the heterogeneity of the labeled and nonlabeled material (Table 3).

#### 3.2.3 Metabolite profiling

In plasma, napabucasin and M1 were the most abundant components that accounted for 23.2% and 22.5% of plasma TRA, respectively. Four minor metabolites were identified (M2, M3, M4, and M6), but accounted for approximately ≤7.0% of TRA in plasma (Table 4). Cumulatively, napabucasin and quantified metabolites accounted for approximately 72% of TRA in plasma, suggesting the presence of numerous radiolabeled metabolites that were below the 1% limit for quantitation.

**TABLE 4:** Napabucasin and its proposed metabolites (≥1% TRA) identified in plasma, urine, and feces

Metabolite component designation	Proposed identification	Matrix		
		Plasma^a^	Urine^b^	Feces^b^
Napabucasin + quantified metabolites		~72%	19.5%^c^	55.9%
Napabucasin		23.2%	2.92%	54.9%
M1	Dihydro‐napabucasin (Hydroxy BBI608)	22.5%	4.5%	0.982%
M4	Dihydro‐napabucasin sulfate	6.5%	0.357%	—
M2	Dihydroamino‐napabucasin	6.3%	—	—
M6	Dihydro‐napabucasin glucuronide	4.5%	3.66%	—
M3	Dihydro‐napabucasin sulfate	3.7%	0.109%	—
M35	Unidentified	3.6^d^	—	—
M34	Unidentified	1.2^d^	—	—
M8	Tetrahydro‐napabucasin sulfonyl glucuronide	—	2.82%	—
M10	Tetrahydro‐napabucasin sulfonyl glucuronide	—	1.83%	—
M16	Tetrahydro‐napabucasin sulfonyl glucuronide	—	1.01%	—

Abbreviations: AUC, area under the curve; MS, mass spectrometry.

^a^ Metabolite levels in plasma were measured from the across‐subject AUC plasma pool by accelerator MS.

^b^ Metabolite levels in urine and feces were measured from individual subject AUC pools by liquid chromatography‐MS.

^c^ Napabucasin and identified/characterized metabolites accounted for a mean of 19.5% of the dose, whereas unidentified metabolites accounted for a mean of 1.90% of dose.

^d^ M34 and M35 were quantified by accelerator MS, but no structural identification could be elucidated due to the lack of discernable molecular ions or product ion spectra.

In urine, napabucasin was a minor component and accounted for a mean 2.92% of the initially administered dose across subjects (range: 0.44%–8.6%). Identified metabolites >1% of the initially administered dose across individual subjects included M1, dihydro‐napabucasin glucuronides (M6 and M21), dihydro‐napabucasin di‐glucuronide (M11), tetrahydro‐napabucasin sulfate and glucuronide bi‐conjugates (M8, M10, and M16), tetrahydro‐napabucasin glucuronide conjugate (M20), and coeluting glucuronide conjugates (M26/M27). In addition to the unchanged parent, M1, dihydro‐napabucasin glucuronide (M6), and tetrahydro‐napabucasin sulfate and glucuronide bi‐conjugates M8, M10, and M16 were, overall, minor urinary metabolites that accounted for means of approximately 4.5%, 3.7%, 2.8%, 1.8%, and 1% of the initially administered dose, respectively, across subjects. In addition, three identified/characterized trace (<1% of initial dose), one unidentified minor (<10% of initial dose), and 15 unidentified trace metabolites were also quantified across individual subjects (Table 4). Cumulatively, napabucasin and identified/characterized metabolites accounted for a mean of approximately 20% of the initial dose, whereas unidentified metabolites individually and cumulatively accounted for a mean of less than 2% of the initial dose across all subjects.

In feces, napabucasin was the primary component and accounted for a mean of 54.9% (range: 15%–104%) of the initial dose. M1 was the sole fecal metabolite, accounting for a mean of <1% of the initial dose; it was undetected in two subjects and accounted for <2% of the initial dose in the remaining subjects (Table 4). Napabucasin and M1 cumulatively accounted for a mean of approximately 56% of the initial dose (range: 17%–104%).

### 3.3 Safety

All eight subjects (100%) experienced at least one treatment‐emergent AE (TEAE; Table 5). All TEAEs were mild in severity and the majority (93%; 27/29) were considered related to napabucasin. The most commonly reported TEAEs were gastrointestinal disorders, with seven subjects (87.5%) reporting a total of 21 events. The most frequently reported AEs related to napabucasin were chromaturia (*n* = 7, 87.5%), diarrhea (*n* = 4, 50%), and infrequent bowel movements (*n* = 4, 50%) (Table 5). Four subjects reported diarrhea (a total of 12 episodes) that started at ~4.5–5.0 h post‐dose and lasted up to 11 days. Eight instances were considered definitely related to napabucasin and four were considered possibly or unlikely related. All episodes were mild in severity and resolved without treatment. There were no severe AEs, serious AEs, or deaths during the study, and no subject discontinued this study due to an AE. There were no clinically significant laboratory, vital sign, electrocardiogram, or physical examination changes.

**TABLE 5:** Frequency of TEAEs

	TEAEs		Treatment‐related TEAEs	
	Subjects, *n* (%)	Events	Subjects, *n* (%)	Events
All TEAEs	8 (100)	29	8 (100)	27
Gastrointestinal disorders	7 (87.5)	21	7 (87.5)	19
Diarrhea	4 (50.0)	12	4 (50.0)	11
Infrequent bowel movements	4 (50.0)	5	4 (50.0)	5
Abdominal pain	1 (12.5)	1	1 (12.5)	1
Constipation	1 (12.5)	1	1 (12.5)	1
Feces soft	1 (12.5)	1	1 (12.5)	1
Oral discomfort	1 (12.5)	1	0	0
Chromaturia	7 (87.5)	8	7 (87.5)	8

Abbreviation: TEAE, treatment‐emergent adverse event.

## 4 DISCUSSION AND CONCLUSIONS

The objective of this study was to characterize the absorption, metabolism, and excretion of a single 240‐mg oral dose of napabucasin in healthy male subjects. Napabucasin was absorbed into the systemic circulation and was metabolized to yield M1 as the sole major circulating metabolite, which has 12.57‐fold less activity than napabucasin [Data on file, Sumitomo Dainippon Pharma Oncology, Inc.]. Systemic exposure to napabucasin was higher than M1, but both compounds had similar t_1/2_ and CL_R_ values, suggesting that M1 formation was rate limiting. The elimination of napabucasin was mainly via feces (57.2%), followed by urinary excretion (23.8%). In urine, the abundance of napabucasin metabolites and the minor abundance of napabucasin suggest that napabucasin eliminated through the kidneys was cleared by reductive metabolism and, to a lesser extent, by renal elimination. A single oral 240‐mg dose of napabucasin containing approximately 100 µCi ^14^C‐napabucasin was generally well tolerated when administered to healthy male subjects in this study.

**FIGURE 5:**
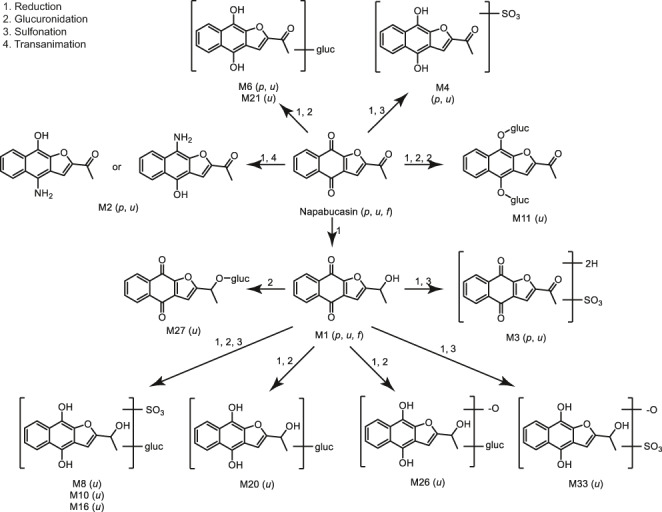
Proposed biotransformation pathways of napabucasin in humans. The metabolite profiling results showed that napabucasin was extensively metabolized to produce 30 metabolites, all of which were found in urine, five in plasma, and one in feces. Based on these results, we propose a biotransformation pathway of napabucasin in humans: reduction of the acetyl side‐chain and/or the naphthalene dione moiety is the exclusive primary biotransformation pathway, with glucuronidation, sulfonation, and, to a lesser extent, transamination as abundant secondary routes of metabolism. f, feces; p, plasma; u, urine

The results of this study show that napabucasin was extensively metabolized to produce 30 metabolites, all of which were found in urine, five in plasma, and one in feces. Based on these findings, we propose the biotransformation pathway of napabucasin in humans illustrated in Figure 5. Reduction in the acetyl side‐chain and/or the naphthalene dione moiety is the exclusive primary biotransformation pathway, with glucuronidation, sulfonation, and, to a lesser extent, transamination as abundant secondary routes of metabolism. A greater, detailed description of this proposed pathway is provided in Figure 5. Reduction of napabucasin on the acetyl side chain produces dihydro‐napabucasin, M1, as a major circulating and excreted metabolite. M1 was abundant in plasma; the sole (trace) metabolite in feces, and was a minor component in urine. Glucuronide conjugation of the resulting hydroxyethyl side chain yields the trace urinary metabolite M27. Reduction in M1 with secondary conjugation yields multiple metabolites mostly found in urine, including sulfate conjugates M3 (also a minor metabolite in plasma) and M33, glucuronide conjugates M20 and M26, and sulfate‐glucuronide di‐conjugates M8, M10, and M16. Reduction of napabucasin on the naphthalene dione moiety forms multiple secondary plasma and urinary metabolites. Reduction with secondary glucuronidation yields dihydro‐napabucasin glucuronides M6 and M21 as a minor plasma and urinary metabolite and as a trace urinary metabolite, respectively. Glucuronide conjugation of both naphthalene diols produces dihydro‐napabucasin di‐glucuronide conjugate M11. Sulfonation of the naphthalene diol yields dihydro‐napabucasin sulfate conjugate M4 as a minor plasma metabolite and trace urinary metabolite. Transamination of the naphthalene diol produces the corresponding amine M2 as a minor circulating metabolite.

The findings of this study are consistent with those from previous studies. In humans, napabucasin was primarily excreted in feces and no new major or disproportionate metabolite was quantified, which aligned with the findings from preclinical studies. Oral administration of ^14^C‐napabucasin resulted in near‐complete elimination (>95%) via the renal as well as biliary/fecal route in rats and mainly via the biliary/fecal route in dogs, within 72 and 24 h, respectively [Data on file, Sumitomo Dainippon Pharma Oncology, Inc.]. However, direct biliary elimination of unchanged napabucasin has not been evaluated. In vitro metabolic studies of ^14^C‐napabucasin with cryopreserved hepatocytes from rats, dogs, rabbits, and humans showed that napabucasin metabolites were qualitatively similar between species [Data on file, Sumitomo Dainippon Pharma Oncology, Inc.]. The main metabolites from rat, rabbit, and dog hepatocytes were M4 (28.0%), M1 (33.9%), and M8 (30.0%), respectively [Data on file, Sumitomo Dainippon Pharma Oncology, Inc.]. M1 was the main metabolite from human hepatocytes [Data on file, Sumitomo Dainippon Pharma Oncology, Inc.].

A single 240‐mg dose of napabucasin led to mild AEs in healthy male subjects in the current study. Safety events were predominately gastrointestinal disorders (e.g., diarrhea), consistent with the observations in other napabucasin clinical trials.^8^, ^17^, ^18^, ^22^ Mild chromaturia in this study (87.5% of subjects) was more frequent than reported in studies that assessed napabucasin in colorectal cancer (29/90 patients [21%])^18^ or combined with paclitaxel in gastric and gastro‐esophageal junction adenocarcinoma (47/357 patients [13.17%]).^16^, ^23^

A limitation of this study was the inability to homogeneously mix the radiolabeled compound with the nonlabeled semisolid suspension due to limited solubility. This lack of homogeneous drug formulation likely resulted in the observed differences in absorption between the radioactive and unlabeled material among subjects, and in the ratio of plasma napabucasin or M1 (based on cold assay) to TRA being >1 in some subjects (based on C_max_ and AUC_last_). It was, therefore, not possible to conclude whether the circulating radioactive material was predominantly parent and M1 or other circulating metabolites by comparing the radioactive and nonradioactive profiles. Additionally, the mean TRA recovery was 81.1%, although the discharge criteria specified >90% recovery unless 12 days had elapsed. However, although commonly used as a discharge criterion, 90% is arbitrary, as was the maximum stay required in this study (12 days) if discharge criteria were not met.

This study showed that napabucasin was absorbed following oral dosing and underwent extensive metabolism to yield M1 as the sole major circulating metabolite. Most of the administered radioactive dose was eliminated via the fecal route and to a lesser extent via urine. A single oral 240‐mg dose of napabucasin was generally well tolerated in healthy male subjects.

## 5 DATA SHARING AND DATA ACCESSIBILITY STATEMENT

Certain of the underlying data remains confidential, non‐public information and is proprietary to the Company. Access to such confidential information upon request remains at the sole discretion of the Company, and will require the requestor enter into an acceptable confidentiality agreement.

## CONFLICT OF INTEREST

XD, MDK, and MH are employees of Sumitomo Dainippon Pharma Oncology, Inc. MLH is a former employee of Moderna, Inc. JEB, NCJEE, NR, and BTG are employees of Covance, Inc. (CRU).

## AUTHOR CONTRIBUTIONS

Conceptualization: X. Dai, M. D. Karol, M. Hitron, M. L. Hard, J. E. Blanchard. Supervision: X. Dai, M. D. Karol, M. Hitron. Project Administration: X. Dai. Resources: X. Dai, J. E. Blanchard, N. C. J. E. Eraut, N. Rich, B. T. Gufford. Investigation: X. Dai, J. E. Blanchard. Formal analysis: X. Dai, M. Hitron, N. C. J. E. Eraut, N. Rich, B. T. Gufford. Writing – original draft and review & editing: X. Dai, M. D. Karol, M. Hitron, M. L. Hard, J. E. Blanchard, N. C. J. E. Eraut, N. Rich, B. T. Gufford.

## Supporting information

Table S1
