# Supplementary material for: Mass balance and pharmacokinetics of an oral dose of 14C‐napabucasin in healthy adult male subjects
Source: Pharmacol Res Perspect. 2021 Feb 11;9(1):e00722. doi: 10.1002/prp2.722 (PMC7878185; doi:10.1002/prp2.722)
Supplement: Supplementary file 1 — Table S1 [file PRP2-9-e00722-s001.docx]

**Supplemental TABLE 1 Accuracy and precision of napabucasin and M1 measurements**

|  | **Plasma** | | **Urine** | |
| --- | --- | --- | --- | --- |
|  | **Napabucasin**  3 runs, *n*=5–6/run | **M1**  3 runs, *n*=6/run | **Napabucasin**  3 runs, *n*=5–6/run | **M1**  3 runs, *n*=6/run |
| Intra-run |  |  |  |  |
| Bias | -8.0–16.6% | -11.0–20.0% | -17.2–0.8% | -4.2–15.0% |
| CV | 0.9–46.7% | 1.8–12.6% | 0.9–29.3% | 1.3–8.4% |
| Inter-run |  |  |  |  |
| Bias | 4.5–8.4% | -8.0–9.0% | -10.9– -2.7% | -1.7–9.0% |
| CV | 2.6–28.2% | 3.0–13.0% | 4.1–16.5% | 4.8–6.2% |

Quality control samples were run for assay validation.

CV, coefficient of variation.
